# Exposure to gestational diabetes mellitus in utero impacts hippocampal functional connectivity in response to food cues in children

**DOI:** 10.21203/rs.3.rs-3953330/v1

**Published:** 2024-03-12

**Authors:** Stephanie Kullmann, Sixiu Zhao, Lorenzo Semeia, Ralf Veit, Shan Luo, Brendan Angelo, Ting Chow, Andreas Birkenfeld, Hubert Preissl, Anny Xiang, Kathleen Page

**Affiliations:** University of Tuebingen; University of Tubingen; University of Tubingen; University of Tubingen; USC; Keck School of Medicine, University of Southern California; Kaiser Permanente Southern California; Universitätsklinikum Tübingen; University of Tübingen/Helmholtz Center Munich; Kaiser Permanente Southern California

## Abstract

**Objectives:**

Intrauterine exposure to gestational diabetes mellitus (GDM) increases the risk of obesity in the offspring, but little is known about the underlying neural mechanisms. The hippocampus is crucial for food intake regulation and is vulnerable to the effects of obesity. The purpose of the study was to investigate whether GDM exposure affects hippocampal functional connectivity during exposure to food cues using functional magnetic resonance imaging.

**Methods:**

Participants were 90 children age 7–11 years (53 females) who underwent an fMRI-based visual food cue task in the fasted state. Hippocampal functional connectivity (FC) was examined using generalized psychophysiological interaction in response to high-calorie food versus non-food cues. Food-cue induced hippocampal FC was compared between children with and without GDM exposure, while controlling for possible confounding effects of age, sex and waist-to-hip ratio.

**Results:**

Children with GDM exposure exhibited stronger hippocampal FC to the insula and striatum (i.e., putamen, pallidum and nucleus accumbens) compared to unexposed children, while viewing high caloric food cues.

**Conclusions:**

Intrauterine exposure to GDM was associated with higher food-cue induced hippocampal FC to reward processing regions. Future studies with longitudinal measurements are needed to clarify whether increased hippocampal FC to reward processing regions may raise the risk of the development of metabolic diseases later in life.

## 1. Introduction

Gestational diabetes mellitus (GDM) is traditionally defined as glucose intolerance with first-time diagnosis during pregnancy (1). It develops in approximately 10% of pregnancies, making it one of the prevalent complications during gestation (2). Intrauterine exposure to GDM increases the risk of developing obesity in offspring (2). It is not yet clear which factors might drive these conditions later in life, but early neurodevelopmental processes appear sensitive to intrauterine hyperglycemia, hyperinsulinemia and neuroinflammation caused by maternal overnutrition, including hyperglycemia (3, 4). Furthermore, intrauterine exposure to GDM may lead to increased food intake, which is regulated by multiple brain regions (5).

The hippocampus is believed to influence food intake by integrating learned experience (food-related episodic memories, associations, incentive information) with interoceptive signals of nutritive state, as well as visual, gustatory, and olfactory cues (for review, see (6)). Animal models and behavioral studies in humans suggest that even a brief exposure to a diet rich in dietary fat and sugar can impair hippocampal-dependent learning and memory (7, 8). Furthermore, behavioral data in healthy humans showed that influencing meal memory may reduce or enhance later food intake. For example, recalling the most recent meal reduced subsequent food intake (9), while dividing attention during the meal increased later food intake (10). Furthermore, amnesic patients fail to interpret interoceptive signals (e.g., hunger and satiety) (11). Using fMRI, the hippocampus has been shown to be responsive to the ingestion of sugar, visual food cue exposure, and insulin administration in healthy adults (12, 13). Hippocampal dysfunctions may impair the ability to retrieve memories of meals, detect interoceptive signals, and lead to overeating (for reviews, see (14)). Significantly, data from animals and human suggests the development of the hippocampus is sensitive to GDM exposure (4, 15–17). In animals, intrauterine exposure to diabetes caused decreased neuronal density and reduced synaptic integrity in the hippocampus (4, 15, 16). GDM exposure was also associated with reduced thickness in the left hippocampus in children (17).

Neural food cue reactivity has been used in children to evaluate the neural basis of appetite control (18, 19). Children and adolescents with obesity exhibited higher neural responses to palatable food images in the reward-related regions, including the striatum, insula, amygdala, and hippocampus (20, 21). Waist circumference, rather than BMI, was associated with higher hippocampal activation during high caloric food cue exposure (22). Studies have demonstrated functional coupling between the hippocampus and multiple brain regions involved in reward processing (23, 24). Yet, evidence from resting-state fMRI studies suggests that the functional connectivity (FC) of the hippocampus to reward-related regions is altered by obesity in both children and adults (25–29). Specifically, children with obesity exhibited lower hippocampal FC to the orbitofrontal cortex and striatum (25, 26), while adults with obesity showed higher hippocampal FC to the striatum and lower to the anterior cingulate cortex, compared to individuals with normal weight (27–29). The discrepancy could be due to the developmental trajectory of the hippocampus (30). Unlike examining intrinsic networks during resting-state, task-based functional connectivity during a food cue task explores the brain’s connectivity during exposure to food cues. During the presentation of appetizing food cues, evidence suggests that hippocampal FC to the orbitofrontal cortex, dorsomedial prefrontal cortex and dorsolateral prefrontal cortex increases with BMI in adults (31). Nevertheless, functional coupling of the hippocampus to other brain regions during exposure to visual food cues in GDM-exposed compared to unexposed children remains unexplored.

The current study investigates the relation between GDM exposure and FC of the hippocampus in children. We examined task-based FC of the bilateral hippocampus in children with and without GDM exposure using generalized psychophysiological interaction (gPPI) in response to visual food cues (high-calorie food minus non-food) in the BrainChild Cohort (5, 18). Prior studies (20, 21, 25–29, 31) indicate higher food-cue-induced neural reactivity of reward regions and alterations in hippocampal FC in children with obesity. Hence, we hypothesized that hippocampal FC is higher to reward-related regions during food cue presentation in children with GDM exposure when compared to children without exposure. Since prior evidence points to distinct effects of GDM on the left and right hippocampus (17) in children, we also investigated FC of the left and right hippocampus separately in an exploratory analysis.

## 2. Methods

### 2.1 Participants

Participants included 112 children from the larger BrainChild study assessing the impact of exposure to GDM *in utero* on neural and endocrine systems underlying risk for obesity and diabetes (32). The BrainChild study included typically developing children aged 7–11 years recruited from Kaiser Permanente Southern California (KPSC) (5, 18). Inclusion criteria included KPSC’s electronic medical records, which documented maternal GDM or normal glucose tolerance during pregnancy, uncomplicated singleton birth, and children with no history of medical/psychiatric disorders or taking medicines affecting metabolism. The institutional review board at both KPSC (# 10282) and University of Southern California (USC) (# HS-14–00034) approved this study. Parents and children were provided with written informed consent and informed child assent prior to the study. Twenty-two participants were excluded due to excessive movement, image artifacts, or the presence of brain lesions. The final analyses included a total of 90 participants.

### 2.2 Maternal GDM exposure

GDM during pregnancy was determined based on one of the following laboratory plasma glucose values during pregnancy: 1) plasma glucose values ≥ 200 mg/dL from a 50 g 1-hr glucose challenge test, 2) at least two plasma glucose values meeting or exceeding the following values on either the 75 g 2-hrs or 100 g 3-hrs oral glucose tolerance test: fasting, 95 mg/dL; 1 h, 180 mg/dL; 2 h, 155 mg/dL; and 3 h, 140 mg/dL (33).

### 2.3 Study procedures

The data for this study were collected over two visits conducted after a 12-h overnight fast. The first visit consisted of metabolic phenotyping, including assessments of anthropometric measures. The second visit was a neuroimaging visit, including functional magnetic resonance imaging (fMRI) measurement during a food cue task after the overnight fast.

### 2.4 First visit: Anthropometric measurement

During the first visit, anthropometric data, including height, weight, waist and hip circumferences of both the mother and child, tanner stage of child were collected at the Clinical Research Unit of the USC Diabetes and Obesity Research Institute as previously reported (32). Specific to children, BMI z-scores (BMI-z) were calculated using the Center for Disease Control (CDC) guidelines (34).

### 2.5 Second visit: MRI measurement

After the overnight fast, fMRI measurements of the children were performed at the USC Dana and David Dornsife Neuroimaging Center. Children first underwent training on a mock scanner, after which they were imaged in a 3T MRI scanner. All children were scanned between 8 and 10 am following a 12-h overnight fasting. They completed a visual food cue task in the scanner (For more details, see (18)). Briefly, children were presented high-calorie food (e.g., ice cream) and non-food (e.g., pencils) pictures and instructed to watch the pictures attentively. The stimuli were selected based on pilot studies of children’s ratings of familiarity and appeal of the food and non-food items. And, the food and non-food Items were also selected to include similar characteristics such as contrast, salience, color, shape and complexity. A total of 12 blocks of stimuli were included. Each block included three images and each image was displayed for 4s with 1s waiting between pictures. The sequence of the blocks was randomized. The food cue task lasted 196 s in total.

### 2.6 Image acquisition and preprocessing

The imaging was conducted on a Siemens MAGNETOM Prismafit 3T MRI scanner with a 20-channel head coil. Functional images were obtained using a 2D single-shot gradient echo planar imaging sequence with the following parameters: repetition time (TR) = 2000 ms; echo time = 25 ms; flip angle = 85°; voxel resolution 3.4 × 3.4 ×4 mm^3^; 32 axial slices. A high-resolution structural image was also acquired at 1×1×1 mm^3^ resolution. For more details, see publication (18).

The preprocessing of the fMRI data was performed using SPM12 (http://www.fil.ion.ucl.ac.uk/spm). Slice timing and realignment were performed for each fMRI time series. Movement criteria was movement > 2° or 2mm in any direction, or mean framewise displacement of more than 0.3 mm. The resulting mean functional image and the structural image was coregistered. Unified segmentation was performed to the anatomical image and normalization parameters were estimated. Then, these parameters were applied to the functional images and normalized into MNI space, using the same method applied in our previous paper by Luo et al. (18) and in other studies (35, 36) with children within the same age range. The data were then smoothed with an 8 mm field-width half-maximum (FWHM) Gaussian kernel. Physiological noise signals in the white matter and cerebrospinal fluid were extracted using Principal Component Analysis (PCA) using the PhysIO toolbox (37).

### 2.7 Region of interest (ROI) definition

**Figure 1:**
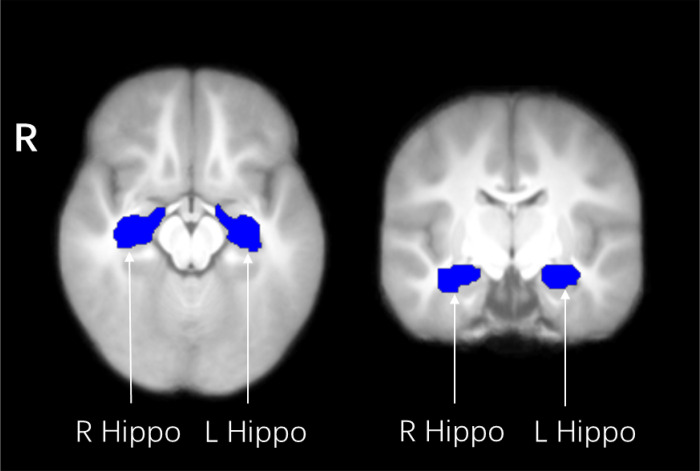
Masks of the hippocampus derived from the AAL atlas 3, overlaid on the average normalized T1 weighted image of the children. Hippo, Hippocampus; L, left; R, right.

To specifically investigate the effect of GDM on the hippocampus FC, we used an anatomical ROI-based approach. Left, right and bilateral ROIs of the hippocampus were created using the AAL atlas 3 (AAL3, https://www.oxcns.org) (Fig. 1).

### 2.8 Subject level analysis

For each participant, the brain response to high-calorie food and non-food images were convolved with a canonical hemodynamic response function, and then added to the General Linear Model (GLM). The six motion parameters, and three components each of the white matter and cerebrospinal fluid signals extracted by PCA were also included in the GLM as confounds. High-pass filtering was applied (128 s).

### 2.9 Hippocampal functional connectivity during food cue task

Task-based FC between anatomical seed region of the hippocampus (i.e., bilateral hippocampus) and all other brain voxels was assessed using a generalized psychophysiological interaction (gPPI) approach (https://www.nitrc.org/projects/gppi version 13.1). In an exploratory analysis, FC was assessed for the right and left hippocampus separately in the same way.

Firstly, the time series from the seed region were extracted. Secondly, the PPI interaction terms were generated for food and non-food stimuli according to the time series. Finally, FC of the seed region was computed for food and non-food stimuli for each participant.

### 2.10 Second level analysis

To evaluate intrauterine exposure to GDM on food-cue induced hippocampal FC, the gPPI contrast maps of *food minus non-food* were entered into a second-level two-sample t-test model with the GDM exposure (GDM vs. Non-GDM) as grouping factor. Age and sex were included in the model as covariates due to their potential effects on hippocampal structure and function (17, 24).WHR rather than BMI has been reported to be positively correlated with higher food-cue reactivity in the hippocampus (22) and we recently reported higher WHR in children with GDM exposure (5). Therefore, waist-to-hip ratio (WHR) was adjusted for possible impact of adiposity.

The statistical parametric maps were thresholded using an uncorrected threshold of *p* < 0.001 and a cluster-level family wise error (FWE) corrected threshold of *p* < 0.05. In addition, small volume correction (SVC) was performed for the insula and striatum (caudate, putamen, nucleus accumbens, pallidum), based on their activation in response to food reward processing and influenced by obesity in children and adolescents (20, 21). The striatal mask and the insular mask were generated based on AAL3 (https://www.oxcns.org) and the wfu pick atlas (https://www.nitrc.org/projects/wfu_pickatlas/). Multiple comparison was implemented for two masks using corrected threshold *p* < 0.025.

## 3. Results

### 3.1 Demographics

The demographics of the 90 participants included in this study are shown in Table 1 (ages 7–11 years, 53 females, 50 GDM exposed), and 89% of children were in Tanner Stage 1. There were no significant differences in children’s age, sex, BMI z-score, or maternal current BMI or maternal prepregnancy BMI among GDM exposed vs. unexposed groups (*p* > 0.05, Table 1). There was a trend towards a higher WHR for children exposed to GDM than unexposed (t (88) = 1.97, *p* = 0.052, Table 1).

### 3.2 Hippocampal task-based functional connectivity in response to food cues

**Figure 2:**
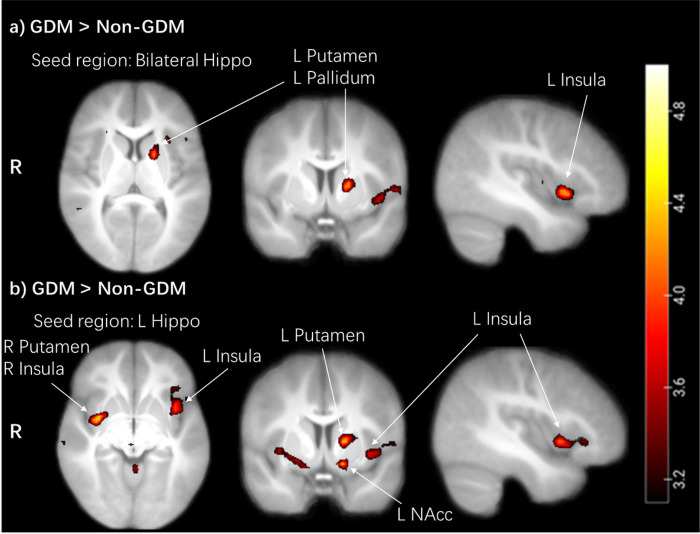
Hippocampal functional connectivity during the food-cue task. a) children with GDM exposure showed higher FC between bilateral hippocampus and left insula, left putamen/pallidum. b) children with GDM exposure showed higher FC between left hippocampus and the bilateral putamen, insula, and left NAcc. The cluster of the right putamen extended to the right insula. The cluster of left putamen extended to the left pallidum. Color map corresponds to T values (*p* < 0.001 uncorrected for display) overlaid on the normalized average T1 weighted image of the children. Hippo, hippocampus; FC, functional connectivity; GDM, gestational diabetes mellitus; NAcc, nucleus accumbens; L, left; R, right.

We observed higher FC in children with GDM exposure compared to children without GDM exposure between the bilateral hippocampus and the left insula (*p*_FWE_ = 0.037) and left putamen, which extended to the pallidum (*p*_FWE_ = 0.019, SVC) (Table 2, Fig. 2).

In an exploratory analysis, FC was assessed for the right and left hippocampus separately. In children with GDM exposure compared to children without exposure, we observed higher FC between the left hippocampus and the right putamen (*p*_FWE_ = 0.007), left putamen (*p*_FWE_ = 0.017, SVC), right insula (*p*_FWE_ = 0.017), left insula (*p*_FWE_ = 0.011, SVC), left nucleus accumbens (NAcc, *p*_FWE_ = 0.013, SVC) (Table 2, Fig. 2). The cluster of the right putamen extended to the right insula. The cluster of the left putamen extended to the left pallidum. No group differences were found for the right hippocampus FC.

## 4. Discussion

The current study investigated the relationship between intrauterine GDM exposure and food cue induced hippocampal functional connectivity in children aged 7–11 years in the fasted state. Consistent with our hypothesis, children with GDM exposure compared to unexposed showed higher hippocampal FC to reward processing regions (i.e., putamen, pallidum, NAcc and insular cortex) adjusted for age, sex and adiposity.

We observed higher functional coupling between hippocampus to striatal regions and insula in children with intrauterine GDM exposure compared to children without exposure, primarily driven by the left hippocampus. A prior structural MRI report found reduced left hippocampal thickness in children with GDM exposure compared to unexposed children (17). Therefore, GDM may affect both the structure and function of the hippocampus. Hippocampal neurons interact with other neurons in the mesolimbic system receiving dopamine projections to communicate rewarding properties of environmental stimuli (6, 38). As potent rewards, palatable foods can trigger associations with reward and motivational behaviors that potentially could lead to overeating and eventual weight gain (38). These food cues tend to evoke heightened memories and mental simulations of consumption in children (39). Higher activation in the striatum and insula in response to food images were observed in children and adolescents with obesity compared to their healthy-weight peers (19–21). In the resting state, higher striatal and insular network FC was also linked to eating in the absence of hunger, food craving, disinhibited eating, weight gain and obesity in both children and adults (40–43). Current findings on FC between the hippocampus and striatum are inconsistent. Adolescents with obesity exhibited lower resting-state hippocampal FC to the dorsal striatum (26), while adults exhibited the opposite pattern (27, 28). Furthermore, FC between the hippocampus and striatum was lower during milkshake consumption in adults with obesity compared to those with healthy-weight (44). According to recent studies, resting-state FC of the hippocampus to the striatum increased with BMI and was associated with higher emotional eating scores and weight gain in adults (27, 28). A meta-analysis also indicated that the hippocampus-striatum connection may play a role in craving and the formation of habits associated with obesity (45). Whether higher hippocampal connectivity to the striatum and insula in children with GDM exposure are the predictors of the development of obesity later in life is currently unknown. A longitudinal study and follow-up into adolescent and adulthood will be needed to reveal the impacts.

Of note, our study points to a distinct effect of intrauterine GDM exposure on the brain, rather than obesity itself. Children exposed to GDM exhibited higher hippocampal FC to striatal regions and the insula when compared to children without exposure, even though there were no group differences in BMI-z score at this young age. These results align with animal studies (4, 15, 16) and provide evidence to support the hypothesis that prenatal exposure to diabetes might result in changes in brain pathways. These changes, in turn, may contribute to the increased risk of weight gain and obesity in affected children. Interestingly, previous studies suggest that hyperactivity in the brain’s reward system in response to rewards might be a susceptibility factor for developing obesity (46, 47). For example, adolescents of healthy-weight but with a high risk for developing obesity (due to parents with excess weight) showed greater striatum and insula activation in response to rewards and higher ad libitum intake, compared to their low-risk counterparts (46, 48). Similarly, our previous study showed that children exposed to GDM had higher daily energy intake (5). However, future studies with longitudinal measurements are necessary to evaluate whether hippocampal changes in FC result in weight gain and raise the risk of developing obesity later in life.

Our study includes some limitations. Given the limited size of our sample, each subgroup, based on GDM exposure, included a relatively small number of subjects. Food intake was not assessed, and future studies are necessary to provide a more detailed understanding how the observed functional alterations in the hippocampus are related to behavior. Moreover, longitudinal data are needed to examine the association between functional alterations in the hippocampus and future weight gain in children.

## 5. Conclusion

The current study suggests that intrauterine exposure to GDM alters hippocampal food cue processing in children. During palatable food pictures presentation, children with GDM exposure exhibited higher hippocampal connectivity to reward processing regions. These alterations may be associated with a potential risk for future weight gain. Longitudinal research is required to determine if stronger hippocampus-reward system connectivity during exposure to food cues leads to future weight gain and a higher likelihood of metabolic disorders, including obesity.

## Figures and Tables

**Table 1:** Demographics*

	Overall	Non-GDM	GDM	t/Z	*p*
	90	N = 40	N = 50		
**Children**					
Age (years)	8.23 (7.82, 9.08)	8.61 (7.75, 9.67)	8.14 (7.85, 8.63)	1.406	0.30
Sex					0.13
Female	53 (58.9%)	20 (50%)	33 (66%)		
Male	37 (41.1%)	20 (50%)	17 (34%)		
BMI z-score	0.66 (0.03, 1.67)	0.59 (−0.06, 1.68)	0.76 (0.23, 1.69)	0.285	0.43
WHR	0.87 ± 0.06	0.86 ± 0.06	0.88 ± 0.06	1.969	0.052
**Mother**					
Current BMI (kg/m^2^)	30.27 (26.52, 35.43)	29.58 (25.60, 35.06)	30.71 (26.90, 26.11)	4.921	0.64
Prepregnancy BMI (kg/m^2^)	29.07 (25.23, 33.22)	29.07 (24.61, 32.98)	28.98 (25.37, 33.51)	2.186	0.61

Abbreviations: BMI, body mass index; GDM, gestational diabetes mellitus; WHR, waist-to-hip ratio; t, statistic for independent-samples t-test; Z, statistic for Mann-Whitney test for data with skewed distribution.

* For continuous variables, normally distributed data (WHR) were described as mean ± standard deviation (SD); data from skewed distribution were described by the median (Q1, Q3); Categorical variable was described as N (%), *p* value was calculated using Chi-square test.

**Table 2:** Hippocampus task-based functional connectivity in response to high-caloric food versus non-food cues adjusted for age, sex and WHR

	MNI coordinates							
	Brain region	Hemi	x	y	z	Peak t	Cluster size	*p*_FWE_
Seed region	GDM > Non-GDM							
Bilateral Hippo								
	Insula	L	−42	14	−7	4.31	59	0.037
	Pallidum/ Putamen	L	−15	2	2	4.29	23	0.019^SVC^
Hippo L								
	Putamen	R	36	−1	−4	4.87	91	0.007
	Insula	R	39	−1	−4	4.20	74	0.017
	NAcc	L	−15	5	−13	4.41	5	0.013^SVC^
	Putamen/ Pallidum	L	−18	5	5	4.32	30	0.017^SVC^
	Insula	L	−42	8	−4	4.42	20	0.011^SVC^
Hippo R								
	No differential activation							
	Non-GDM > GDM							
	No differential activation							

Abbreviations: FWE, family wise error; GDM, gestational diabetes mellitus; Hemi, hemisphere; NAcc, nucleus accumbens; L, left; R, right; p value FWE corrected using whole-brain cluster correction; SVC *p*_FWE_ small volume corrected for ROIs. No group differences were found for the right hippocampus.

## Data Availability

Data is available upon reasonable request from K.A.P.
